# Image Reconstruction Using Analysis Model Prior

**DOI:** 10.1155/2016/7571934

**Published:** 2016-06-09

**Authors:** Yu Han, Huiqian Du, Fan Lam, Wenbo Mei, Liping Fang

**Affiliations:** ^1^School of Information and Electronics, Beijing Institute of Technology, Beijing 100081, China; ^2^Beckman Institute for Advanced Science and Technology, University of Illinois at Urbana-Champaign, Urbana, IL 61801, USA; ^3^School of Mathematics, Beijing Institute of Technology, Beijing 100081, China

## Abstract

The analysis model has been previously exploited as an alternative to the classical sparse synthesis model for designing image reconstruction methods. Applying a suitable analysis operator on the image of interest yields a cosparse outcome which enables us to reconstruct the image from undersampled data. In this work, we introduce additional prior in the analysis context and theoretically study the uniqueness issues in terms of analysis operators in general position and the specific 2D finite difference operator. We establish bounds on the minimum measurement numbers which are lower than those in cases without using analysis model prior. Based on the idea of iterative cosupport detection (ICD), we develop a novel image reconstruction model and an effective algorithm, achieving significantly better reconstruction performance. Simulation results on synthetic and practical magnetic resonance (MR) images are also shown to illustrate our theoretical claims.

## 1. Introduction

Sparse sampling theory [1–3] plays a key role in a broad spectrum of techniques involved in signal and image processing over the past decade. It states that an unknown signal can be recovered from a small number of random linear measurements given that the signal is sparse. Consider a sparse signal or image, vectorized as **x** ∈ *ℝ* ^*n*^, which has very few nonzero elements in the sense that ‖**x**‖_0_ ≪ *n*, where ‖**x**‖_0_ is the count of the nonzeros in **x**. We expect to reconstruct **x** by solving the following *l*_0_-norm minimization problem,

(1) x^=argminx x0subject  to y=Mx,

where **M** ∈ *ℝ* ^*m*×*n*^  (*m* < *n*) denotes the measurement matrix that produces the measurement vector **y** ∈ *ℝ* ^*m*^. However, this is an NP-hard problem which prompts us to look for alternatives to solve it in an approximate fashion. A popular and effective way is to rewrite (1) as the basis pursuit problem:

(2) x^=argminx x1subject  to y=Mx.

As a matter of fact, this is not the general case since signals or images do not exhibit sparsity directly but have sparse representations in specific transform domains. Broadly speaking, there are two data models for describing signals. The first one is the sparse synthesis model [4, 5], wherein **x** is assumed to admit sparse representation in a fixed dictionary **D** = {**d**_*i*_}_*i*=1_^*d*^ ∈ *ℝ* ^*n*×*d*^. Put differently, **x** = **D** **c** can be viewed as a linear combination of very few dominant atoms from **D**. Thus, the reconstruction process (2) is reformulated as

(3) c^=argminc c1subject  to y=MDc; x^=Dc^.

As we know, a tremendous surge of effort has been devoted to studying the sparse synthesis model, and much progress has been made ranging from theoretical foundations [6, 7] to appealing applications, including denoising [8], inpainting [9], and more. Additionally, a long series of algorithms [10–13] together with provable guarantees [14, 15] have been put forward.

While the synthesis model has gained widespread attention, a similar alternative was proposed modeling signals from an analysis perspective [16–20]. Mathematically, given an analysis operator **Ω** = {**ω**_*j*_}_*j*=1_^*p*^ ∈ *ℝ* ^*p*×*n*^, a signal belonging to the analysis model is supposed to admit a sufficiently sparse analysis representation **z** = **Ω** **x** ∈ *ℝ* ^*p*^. In particular, we are interested in cases where *p* ≥ *n*, so that various information within **x** can be captured [17]. The commonly used analysis operators include the finite difference [21], overcomplete wavelet transforms [22], and the curvelet transform [23]. Just in contrast to the “sparsity” in the synthesis setting, the analysis model concentrates on the zero-valued coefficients. A fundamental notion measuring the quantity of the zeros is defined as cosparsity

(4) $$\begin{array}{c} l=p-{\left\|\;\Omega x\;\right\|}_{0}. \end{array}$$

The *l*-cosparse analyzed vector **z** is assumed to have *l* unknown locations of the zeros, referred to as the cosupport

(5) $$\begin{array}{c} \Lambda =\left\{\;j\;|\;\langle \;\omega_{j},x\;\rangle =0,\;\;j\in \left\{\;1,\ldots ,p\;\right\}\;\right\}. \end{array}$$

Recent studies have theoretically shown that the analysis model has its own advantage over the synthesis one [17]. Moreover, adopting the analysis model leads to a collection of successful applications, such as denoising [23, 24], inpainting [25], and medical imaging [26, 27]. Models mentioned above simply exploit the (co)sparsity prior that is implicit in signals or images. However, (co)sparsity alone is insufficient for making reasonable inferences from these models, and the minimum measurement requirement for reconstruction is improved limitedly. Obviously, the trade-off between (co)sparsity and measurements can be further improved by imposing* a priori* knowledge. A common way to incorporate prior knowledge is through the use of the signal support, which has been extensively studied in the synthesis context [28–30]. However, relatively little research has been devoted to imposing prior knowledge in the analysis sense. In this work, we wish to investigate the consequences of exploiting analysis model prior. We concentrate on image reconstruction given the* a priori* cosupport knowledge. First, we formulate the reconstruction problem in terms of the analysis operator **Ω** in general position, which means that every set of *n* rows from **Ω** are linearly independent [17]. In such a case, we derive the minimum number of measurements for uniquely determining a cosparse solution to the inverse problem **y** = **M** **x**. The resulting number is smaller than that of the standard analysis model without using prior cosupport. Second, we dive into details of the model associated with the 2D finite difference operator whose rows show significant linear dependencies [17]. We also provide an improved minimum measurement requirement for guaranteeing the uniqueness given the prior cosupport. Having a theoretical foundation for the analysis uniqueness properties, we develop a novel image reconstruction method based on the idea of iterative cosupport detection (ICD). This two-stage scheme proceeds by alternatingly calling its two key components: image reconstruction and cosupport detection. At each iteration, the image is reconstructed using the cosupport knowledge extracted from the previous iteration. After the acquiring of the image estimate, one can identify even better cosupport to be used in the next iteration. Moreover, when performing the cosupport detection, a multidirectional finite difference is considered. Therefore, the detection and the reconstruction parts work together enabling us to gradually obtain reliable cosupport and a reasonable image estimate. Simulation results on synthetic and practical magnetic resonance (MR) images demonstrate the effectiveness and show considerable improvement of the proposed method compared with other regularization based methods. This consequently indicates that, through the use of the analysis model prior, we can achieve a given reconstruction quality with fewer measurements or alternatively obtain a better reconstruction under the same measurement requirement.

The remainder of this paper is organized as follows. In Section 2, a detailed description of our proposed method is given from two aspects: analysis operators in general position and the 2D finite difference operator. Uniqueness issues are also explored.  Section 3 describes the methods used for comparison and rules for image quality assessment.  Section 4 presents simulation results and discussion which validate our theoretical claims. Finally in Section 5, conclusions are summarized.

## 2. Method

Analysis model prior has been successfully used for many signal processing tasks but has been done with little theoretical justification. In this section, we focus on the analysis-based reconstruction given that the cosupport is known* a priori*. The number of measurements required for guaranteeing the unique reconstruction is proved to be essentially reduced. We first consider the analysis operators in general position.

### 2.1. Analysis Operators in General Position

#### 2.1.1. The Cosupport Is Exactly Known

Consider *l*-cosparse image **x** ∈ *ℝ* ^*n*^ whose cosupport is Λ and a redundant analysis operator **Ω** ∈ *ℝ* ^*p*×*n*^  (*p* ≥ *n*) in general position; namely, every set of *n* rows from {**ω**_*j*_}_*j*=1_^*p*^ are linearly independent [17]. The cosparse representation analysis model where **x** belongs is related to the recently proposed union-of-subspaces model [31–33]:

(6) $$\begin{array}{c} x\in \underset{\left|\Lambda \right|=l}{⋃}W_{\Lambda }, \end{array}$$

which is a union of all the $\left(\begin{array}{c} p\\ l \end{array}\right)$ possible subspaces of dimension *n* − *l*. Here, *𝒲*_Λ_ = Null(**Ω**_Λ_) denotes the null-space of the analysis operator **Ω** indexed by Λ. |Λ| is the cardinality of Λ. The rows associated with Λ, {**ω**_*j*_}_*j*∈Λ_, define the analysis subspace. Remark that removing rows from **Ω** for which 〈**ω**_*j*_, **x**〉≠0 leaves the subspace unchanged.

Consider the aforementioned noiseless linear inverse problem:

(7) $$\begin{array}{c} y=Mx, \end{array}$$

where **M** ∈ *ℝ* ^*m*×*n*^ and **Ω** are assumed to be mutually independent. Provided that the cosupport Λ is exactly known, the cosparse **x** is supposed to satisfy the linear system:

(8) $$\begin{array}{c} \left[\begin{array}{c} y\\ 0 \end{array}\right]=\left[\begin{array}{c} M\\ \Omega_{\Lambda } \end{array}\right]x. \end{array}$$

The fact that (8) identifies a unique **x** is equivalent to the requirement:

(9) $$\begin{array}{c} W_{\Lambda }\cap Null\left(\;M\;\right)=\left\{\;0\;\right\}. \end{array}$$

This requirement indicates that the minimum number of measurements is:

(10) $$\begin{array}{c} m\geq \underset{\Lambda }{max}\;dim\left(\;W_{\Lambda }\;\right)=n-l. \end{array}$$

#### 2.1.2. The Cosupport Is Unknown

Actually, the case we do care about is that we only know the cosparsity level *l*, whereas the cosupport Λ is undetermined. The uniqueness issue in this sense has been explored in [17].

**Lemma 1 .**

Let ⋃_Λ_ *𝒲*_Λ_, |Λ | = *l*, be a union of *l*-cosparse analysis subspaces induced by the analysis operator **Ω**. Then the linear system **y** = **M** **x** admits a unique *l*-cosparse solution if and only if for any |Λ_1_ | , |Λ_2_ | = *l*

(11) $$\begin{array}{c} W_{\Lambda_{1},\Lambda_{2}}\cap Null\left(\;M\;\right)=\left\{\;0\;\right\}, \end{array}$$

where

(12) WΛ1,Λ2WΛ1⊕WΛ2=u ∣ u=x1+x2,  x1∈WΛ1,  x2∈WΛ2.

Clearly, for any **u** ∈ *𝒲*_Λ_1_,Λ_2__, we have cosupp(**u**)⊇Λ_1_∩Λ_2_ and |Λ_1_∩Λ_2_ | ≥ 2*l* − *p*. Therefore, we can conclude that the minimum number in terms of the cosparsity level *l* is

(13) $$\begin{array}{c} m\geq \underset{\Lambda_{1},\Lambda_{2}}{max}\;dim\left(\;W_{\Lambda_{1},\Lambda_{2}}\;\right)=n+p-2l. \end{array}$$

#### 2.1.3. The Cosupport Is Inaccurately Known

More generally, we are considerably interested in the case where the cosupport is inaccurately known. In other words, a superset of the true cosupport is available, which comes up in many applications. Even in the absence of available prior knowledge, one can still extract useful information from the current solution and use it subsequently.

We still assume **x** ∈ *ℝ* ^*n*^ is *l*-cosparse with cosupport Λ. Λ_0_ is the prior cosupport containing small errors denoted by Λ′. Then, the true cosupport of **x** can be expressed as

(14) $$\begin{array}{c} \Lambda =\Lambda_{0}\setminus \Lambda^{\prime }. \end{array}$$

We state the condition which enables us to guarantee the uniqueness of the linear inverse problem (7), namely, the minimum measurement requirement.

**Proposition 2 .**

Assume that **x** ∈ ⋃_Λ_ *𝒲*_Λ_, |Λ | = *l*, is the *l*-cosparse image to be reconstructed. Λ_0_ is the prior cosupport with small error Λ′⊆Λ_0_. Given |Λ_0_ | = *l*_0_ < *p* and |Λ′ | = *l*′ ≪ *l*_0_, the minimum number of measurements for identifying a unique solution to the linear system **y** = **M** **x** is

(15) $$\begin{array}{c} m\geq n+l_{0}-2l. \end{array}$$

**Proof**

As we have seen, the prior cosupport Λ_0_ is not exactly consistent with the true cosupport Λ of **x**; that is, there exists small error Λ′ in Λ. It means that we are supposed to consider the invertibility of **M** over the direct sum *𝒲*_Λ_1_,Λ_2__ of any two subspaces *𝒲*_Λ_1__, *𝒲*_Λ_2__⊆⋃_Λ_ *𝒲*_Λ_. Assume any two *l*-cosparse images **x**_1_ ∈ *𝒲*_Λ_1__, **x**_2_ ∈ *𝒲*_Λ_2__ with corresponding cosupports Λ_1_ = Λ_0_∖Λ_1_′ and Λ_2_ = Λ_0_∖Λ_2_′, respectively. Note that |Λ_1_ | = |Λ_2_ | = *l* and let |Λ_1_′ | = |Λ_2_′ | = *l*′. Then, we have *l* = *l*_0_ − *l*′. Consider any **u** from *𝒲*_Λ_1_,Λ_2__ as defined in (12). The cosupport of **u** is obtained as

(16) cosuppuΛ1∩Λ2=Λ0∖Λ1′∩Λ0∖Λ2′=Λ0∩Λ1′c∩Λ0∩Λ2′c=Λ0∩Λ1′∪Λ2′c=Λ0∖Λ1′∪Λ2′,

which yields

(17) $$\begin{array}{c} \left|\;cosupp\left(\;u\;\right)\;\right|\geq l_{0}-2l^{\prime }. \end{array}$$

Thus, we have

(18) $$\begin{array}{c} dim\left(\;W_{\Lambda_{1},\Lambda_{2}}\;\right)\leq n-\left(\;l_{0}-2l^{\prime }\;\right)=n+l_{0}-2l. \end{array}$$

Consequently, we conclude that the minimum number of measurements

(19) $$\begin{array}{c} m\geq \underset{\Lambda_{1},\Lambda_{2}}{max}\;dim\left(\;W_{\Lambda_{1},\Lambda_{2}}\;\right)=n+l_{0}-2l, \end{array}$$

which completes the proof.

Proposition 2 states that the measurement number required for uniquely identifying the solution to (7) tends to be smaller in the sense that the cosupport is known* a priori*. As *l*_0_ approaches *p*, *m* is equivalent to that of the case without prior knowledge; namely, *n* + *p* − 2*l*. When the prior cosupport selected is consistent with the ground-truth, the minimum measurement number is achieved as *n* − *l*.

### 2.2. Specific 2D Finite Difference Analysis Operator

In this subsection, we would like to investigate the family of finite difference operators on graphs, **Ω**_DIF_ (significantly related to total variation norm minimization [21]), which has been proven successful for sparse signal and image recovery [34–36]. We explore uniqueness issues in the sense that cosupport knowledge is known* a priori*. Due to the fact that this class of analysis operators exhibit strong linear dependencies [17], the theoretical results derived above cannot be applied directly. Our analysis is based upon the work [17]. For ease of notation, we will drop the subscript DIF and simply use **Ω** to represent **Ω**_DIF_ hereafter when there is no ambiguity. Assume that **Ω** is defined on a 2D nonoriented graph with vertices *V* and edges *E*. An edge *e* = (*v*_1_, *v*_2_) connecting two vertices *v*_1_ and *v*_2_ can be regarded as a finite difference. Λ is a subset of *E* and the set of vertices connected by at least one edge in Λ is denoted by *V*(Λ) which is composed of *J*(Λ) connected components. A connected component is a collection of vertices connected to one another by a walk through vertices within Λ. Thus, the dimension of *𝒲*_Λ_ = Null(**Ω**_Λ_) is given by

(20) $$\begin{array}{c} dim\left(\;W_{\Lambda }\;\right)=\left|\;V\;\right|-\left|\;V\left(\;\Lambda \;\right)\;\right|+J\left(\;\Lambda \;\right), \end{array}$$

where |*V* | −|*V*(Λ)| denotes the number of isolated vertices which have distinct values from all the neighbors. Suppose that the known part of the cosupport is denoted by Λ_0_ and the number of the connected components is *J*(Λ_0_)≔*J*. Then, in terms of the cosparsity level *l*, we present a concrete bound for dim(*𝒲*_Λ_), which reveals the minimum measurement number.

**Proposition 3 .**

Let **Ω** be the 2D finite difference analysis operator that computes horizontal and vertical discrete derivatives of an *n* = *N* × *N* image **x**. The measurement matrix **M** is assumed to be mutually independent from **Ω**. For a fixed *l* and the known cosupport Λ_0_ which corresponds to *J* connected components, the equation **y** = **M** **x** admits at most one solution with cosparsity *l* only if

(21) $$\begin{array}{c} m\geq 2n-l-\sqrt{2Jl}+J\geq 2\underset{\Lambda }{max}\;dim\left(\;W_{\Lambda }\;\right). \end{array}$$

Proposition 3 reveals that the measurements required for uniquely determining the cosparse solution to the inverse problem can be reduced given that some part of the cosupport is known. Additionally, the minimum measurement number decreases monotonically as the number of the connected components *J* increases. When *J* = 1, the minimum number of measurements $m\geq 2n-l-\sqrt{2l}+1$, which is equivalent to the case that no cosupport information is available. For completeness, the proof can be found in Appendix.

### 2.3. Proposed Reconstruction Model and Algorithm

#### 2.3.1. Reconstruction Model

Armed with the above-described theoretical analyses, we are now in position to solve the reconstruction problem regularized with cosupport prior. More specifically, we expect to constrain the cosparsity of **x** within the prior cosupport Λ_0_:

(22) x^=argminx ΩxΛ00subject  to y=Mx.

However, as previously mentioned, the *l*_0_-norm involved in the combinatorial minimization program is an NP-hard penalty and thus is not feasible to be solved for practical applications. An effective and widely used alternative is the *l*_1_-relaxation:

(23) x^=argminx ΩxΛ01subject  to y=Mx,

in which the *l*_1_-norm enables promoting high cosparsity in the solution. Its desirable convexity facilitates various computationally tractable algorithms, and much recent progress in the theory of analysis *l*_1_-minimization has been made [37, 38]. In this work, we focus on the analysis *l*_1_-recovery which is given in an unconstrained fashion. The objective function is formulated as a linear combination of the data consistency error and a modified cosparsity-inducing penalty:

(24) $$\begin{array}{c} \hat{x}=\underset{x}{argmin}{\left\|\;y-Mx\;\right\|}_{2}^{2}+\lambda T_{\Lambda }\left(\;x\;\right), \end{array}$$

where *λ* > 0 controls the influence between the fidelity term and the regularization term:

(25) $$\begin{array}{c} T_{\Lambda }\left(\;x\;\right)=\overset{4}{\underset{i=1}{\sum }}{\left\|\;{\left(\;\Omega_{i}x\;\right)}_{\Lambda_{i}}\;\right\|}_{1}. \end{array}$$

Remark that the analysis operator we employed in our method is the 2D finite difference operator involving four directional components; that is, {**Ω**_*i*_}_*i*=1_^4^, respectively, compute discrete derivatives in vertical, horizontal, and two diagonal directions. {Λ_*i*_}_*i*=1_^4^ denote the associated four-direction cosupport sets that are detected iteratively.

#### 2.3.2. Algorithm

Having presented the reconstruction model, we now turn to the question how to effectively solve it. We propose a two-stage algorithm based on the idea of iterative cosupport detection (ICD). The proposed ICD allows extracting the reliable information of the underlying solution and enables achieving a reasonable reconstruction. This two-stage scheme proceeds by alternatingly calling its two components: image reconstruction and cosupport detection. In the reconstruction step, we solve a truncated *l*_1_-minimization problem via conjugate gradient method using the cosupport knowledge obtained from the previous iteration. ICD will terminate if the approximate solution is accurate enough. Otherwise, cosupport detection will be performed in light of this inaccurate reconstruction, thereby yielding a better {Λ_*i*_}_*i*=1_^4^ to be used in the next iteration. Consequently, the detection and the reconstruction parts of the proposed ICD work together enabling us to gradually obtain reliable cosupport and a reasonable image estimate.

It is important to note that the proposed ICD requires reliable cosupport detection. However in most cases, it is hard to completely avoid false detections at each iteration. To this end, we address ways to identify the index sets {Λ_*i*_}_*i*=1_^4^. Actually, the analyzed vectors in each direction {**Ω**_*i*_ **x**}_*i*=1_^4^ are not strictly cosparse but exhibit a strong decay. A conceptually simple detection strategy is to obtain the cosupport in a truncated manner. Without loss of generality, we assume {*I*_*i*_}_*i*=1_^4^ as the four-direction index sets of {*Ω*_*i*_ **x**}_*i*=1_^4^ after sorting in an ascending order. Then, the cosupport set in the *i*th direction is created as

(26) $$\begin{array}{c} \Lambda_{i}={\left\{\;I_{i}\left(\;k\;\right)\;\right\}}_{k=1}^{L}, \end{array}$$

where the truncated length *L* is fixed and specified in advance. Empirically, a relatively stable reconstruction can be achieved provided that *L* is suitably scaled in a certain range. This strategy is easy to implement but has one drawback; that is, the assumption that the true cosupport is included in the inaccurate prior cosupport cannot be well satisfied when *L* is fixed. To alleviate this drawback, we propose another detection strategy which is more adaptive and effective. The cosupport sets {Λ_*i*_}_*i*=1_^4^ are iteratively learned based on thresholding [28]:

(27) $$\begin{array}{c} \Lambda_{i}=\left\{\;j\;|\;\left|\;{\left(\;\Omega_{i}x\;\right)}_{j}\;\right|<\beta_{i}\;\right\}, \end{array}$$

where (**Ω**_*i*_ **x**)_*j*_ represents the *j*th element of **Ω**_*i*_ **x**. The threshold *β*_*i*_ is set as

(28) $$\begin{array}{c} \beta_{i}=\frac{{\left\|\Omega_{i}x\right\|}_{\infty }}{\eta_{i}},\;\left(\eta_{i}>0\right). \end{array}$$

Here, *η*_*i*_ is the threshold parameter selected as an exponential function of the iteration number *t*:

(29) $$\begin{array}{c} \eta_{i}^{t}=w^{t-1}, \end{array}$$

where *w* > 1 is a positive integer. For *t* = 1, we have *β*_*i*_^1^ = ‖**Ω**_*i*_ **x**‖_*∞*_, indicating that no prior cosupport knowledge is exploited in the first iteration. In the sequel, the threshold decreases with the increase of the iteration so that the cosupport size reduces gradually. However, the cosupport in each direction is not strictly decreasing over the iteration, which allows the current detection to include indices within the true cosupport that are excluded from the detected cosupport in previous iterations. This leads to an attractive self-corrected capacity of the cosupport detection. The proposed algorithm, referred to as ICD, is outlined as follows.

**Algorithm 4 (ICD).**

Consider the following:

(1) * Input.* Consider undersampled measurements **y**, cosupport detection parameter *w* (or *L*), regularization parameter *λ*, and maximum iteration number *t*_max_.
(2) * Iteration.* While the stopping criterion is not reached, do the following:
  (i) * Image Reconstruction.* Solve the minimization problem (24) for the image estimate ${\hat{x}}^{t}$ using the cosupport information of the previous iteration {Λ_*i*_^*t*−1^}_*i*=1_^4^.
  (ii) * Cosupport Detection*. Update the cosupport information {Λ_*i*_}_*i*=1_^4^ using the detection criterion equation (27) (or (26)) based on ${\hat{x}}^{t}$.
  (iii) Consider *t* = *t* + 1.
(3) * Output.* The reconstructed image $\hat{x}=x^{t}$.

## 3. Evaluation

To evaluate the performance of the proposed method, we performed simulations on both synthetic and practical MR images. Similar to prior work on CS-MRI [34, 39], we simulated the data acquisition by randomly sampling the 2D discrete Fourier transform coefficients of test images according to the patterns. Thus, the measurement matrix in (24) was defined as the undersampled Fourier transform **F**_**u**_. The number of compressive measurements was measured in terms of the percentage of total number of Fourier coefficients, namely, the sampling ratio (SR). All simulations were carried out under MatLab R2011b running on a PC with a 3.2 GHz processor and 4 GB memory. To assess the effectiveness of the proposed method, we compared it with other potential reconstruction techniques, including (i) SparseMRI [39]: the leading MR image reconstruction approach combining the wavelets and the standard total variation (TV) regularization, regardless of the effect of the wavelets, this approach can be approximately viewed as the proposed method without using cosupport information; thus the contribution of the integration of analysis* a priori* knowledge can be demonstrated; (ii) NLTV [40]: the well-known method based on wavelet sparsity and nonlocal TV penalty; (iii) ISD-TV [28–30]: the method constraining the sparsity of wavelet coefficients over the complement of the known support, which can be regarded as incorporating prior knowledge to the sparse synthesis model; in order to make a fair comparison, we added a TV term; (iv) SDBS-TV [41]: another synthesis-based method combining support knowledge and block-sparse property in the wavelet domain. The proposed method was tested using both truncated and threshold-based strategies, respectively, named as ICD-TR and ICD-TH. We also expected further enhancement by combining our method with the wavelet penalty, named as ICD-WT, which in general solves

(30) $$\begin{array}{c} \hat{x}=\underset{x}{argmin}{\left\|\;y-F_{u}x\;\right\|}_{2}^{2}+\eta_{1}T_{\Lambda }\left(\;x\;\right)+\eta_{2}{\left\|\;\Psi x\;\right\|}_{1}, \end{array}$$

where Ψ denotes the wavelet transform operator and *η*_1_, *η*_2_ > 0. Remark that threshold-based strategy is used for cosupport detection in ICD-WT.

For quantitative evaluation, the reconstruction quality was measured by the relative *l*_2_-norm error (RLNE) which is a standard image quality metric indicating the difference between the reconstruction **x**_*r*_ and the ground-truth **x**_*g*_

(31) $$\begin{array}{c} \zeta_{RLNE}=\frac{{\left\|x_{r}-x_{g}\right\|}_{2}}{{\left\|x_{g}\right\|}_{2}}. \end{array}$$

As for practical MR images containing more fine features, the quality of the reconstruction is quantified by two other metrics. The first one is the high-frequency error norm (HFEN) [42], defined as

(32) $$\begin{array}{c} \zeta_{HFEN}=\frac{{\left\|LoG\left(x_{r}\right)-LoG\left(x_{g}\right)\right\|}_{2}}{{\left\|LoG\left(x_{g}\right)\right\|}_{2}}, \end{array}$$

where LoG is a rotationally symmetric Laplacian of Gaussian filter capturing detailed textures. The filter kernel is of size 15 × 15 pixels and with a standard deviation of 1.5 pixels, the same as that in [42]. The second one is the structural similarity (SSIM) index [43], comparing local patterns of pixel intensities between **x**_*r*_ and **x**_*g*_ that have been normalized for luminance and contrast:

(33) $$\begin{array}{c} \zeta_{SSIM}=\sum_{i}\frac{\left(2\mu_{r,i}\mu_{g,i}+C_{1}\right)\left(2\sigma_{rg,i}+C_{2}\right)}{\left(\mu_{r,i}^{2}+\mu_{g,i}^{2}+C_{1}\right)\left(\sigma_{r,i}^{2}+\sigma_{g,i}^{2}+C_{2}\right)}. \end{array}$$

Here, *μ*_*r*,*i*_ and *μ*_*g*,*i*_ are mean intensities at the *i*th local window of **x**_*r*_ and **x**_*g*_, while *σ*_*r*,*i*_ and *σ*_*g*,*i*_ are the corresponding standard deviations. *σ*_*rg*,*i*_ denotes the covariance and the constants *C*_1_, *C*_2_ are included to avoid instability.

## 4. Results and Discussion

### 4.1. Shepp Logan Phantom

**Figure 1:**
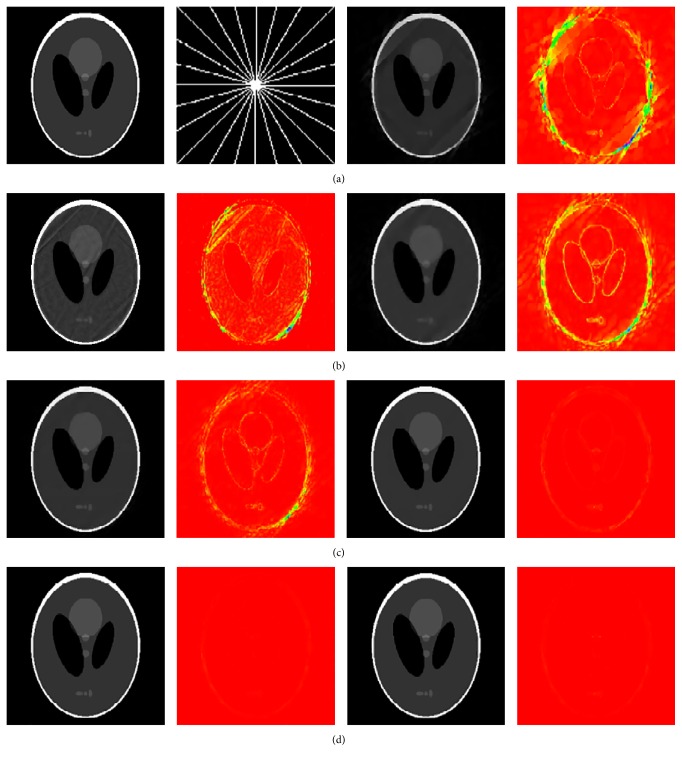
(a) (left to right) shows the original Shepp Logan phantom, the sampling mask, and the reconstructed and error images by SparseMRI. (b) shows the reconstructions by NLTV (left) and ISD-TV (right). (c) shows the reconstructions by SDBS-TV (left) and ICD-TR (right). (d) shows the reconstructions by ICD-TH (left) and ICD-WT (right).

We first tested our method on an ideal example: the Shepp Logan numerical phantom of size 256 × 256. The undersampling scheme we employed is the 2D radial trajectory.  Figure 1 shows the reconstruction results of different methods using 12 radial lines (RL). Reconstruction parameters in (24) were set as follows: *λ* = 5 × 10^−4^ and *L* = 64000 for ICD-TR and *w* = 2 for ICD-TH. We also tested the proposed method using 10 and 11 radial lines. The RLNE comparison results are presented in Table 1. The superiority of the proposed method is clearly seen from reconstructed images and error maps which were obtained by subtracting the reconstructions from the original image (shown at the same scale). The results indicate that methods without using any prior knowledge, such as SparseMRI and NLTV, cause serious artifacts. Methods imposing prior support knowledge in the synthesis sense, including ISD-TV and SDBS-TV, improve the reconstruction quality to a certain extent, but the edges are not well preserved. The proposed ICD significantly improves the reconstruction and is capable of suppressing more artifacts and preserving more details. As for different cosupport detection strategies, ICD-TH performs better than ICD-TR since the threshold-based strategy enables correcting the cosupport adaptively.  Table 2 shows the number of true and false detections of four directional cosupports at each iteration using ICD-TH. It indicates that the number of false detections in each direction decreases gradually until the true cosupport is detected. However, the number of true detections is not necessarily always increasing.

### 4.2. Practical MR Images

**Figure 2:**
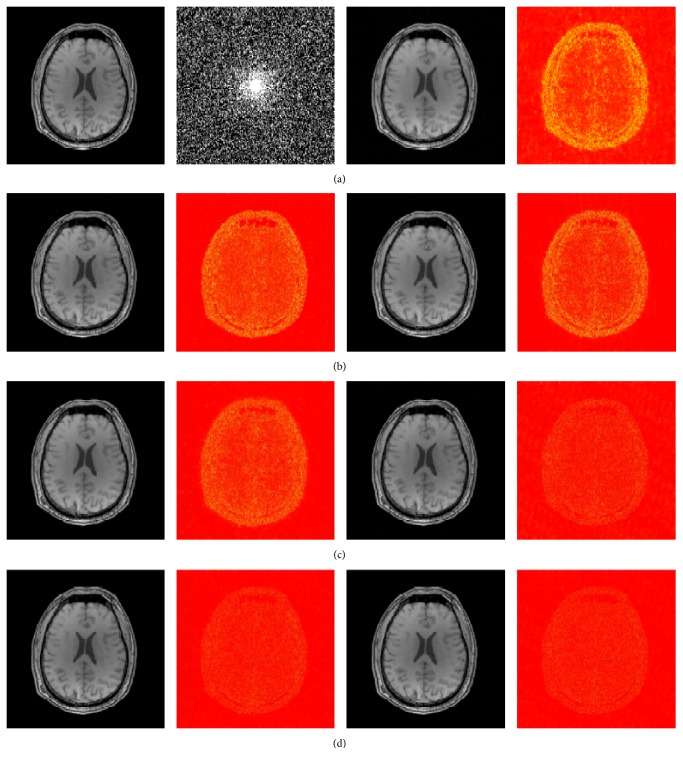
(a) (left to right) shows the original image (Brain-1), the sampling mask, and the reconstructed and error images by SparseMRI. (b) shows the reconstructions by NLTV (left) and ISD-TV (right). (c) shows the reconstructions by SDBS-TV (left) and ICD-TR (right). (d) shows the reconstructions by ICD-TH (left) and ICD-WT (right).

While encouraged by the results on the phantom image, we conducted further simulations on more realistic images to evaluate the practical effectiveness of the proposed method. The CS measurements were generated by undersampling the Fourier coefficients of the fully sampled (ground-truth) MR images whose intensities were normalized to a maximum magnitude of 1. The original T1-weighted image (Brain-1) of size 256 × 256 (courtesy of Professor N. Schuff at the UCSF School of Medicine) and the sampling mask of 30% sampling ratio are shown in Figure 2. The undersampling scheme we used is the variable density random pattern which is widely used in *k*_*y*_-*k*_*z*_ plane for 3D imaging enabling removing the aliasing interference without degrading the image quality [39]. Reconstruction parameters were set as *λ* = 5 × 10^−4^, *L* = 58000 for ICD-TR, and *w* = 5 for ICD-TH. Figure 2 gives the reconstructed images and error maps through different methods, and Table 3 presents the HFEN and SSIM results. The capabilities of our method were also demonstrated under different sampling ratios as presented in Table 1. The reconstruction results show considerable improvement of the proposed method with respect to the subjective visual quality and quantitative indices. It has been noted from the error maps and the HFEN and SSIM results that the image reconstructed by our method has more fine features and is the closest to the ground-truth. The superior performance of ICD can be attributed to the use of the analysis model prior, namely, the cosupport knowledge. Additionally, we observe that the proposed ICD combining the wavelet penalty, ICD-WT, performs slightly better than ICD-TR and ICD-TH. However, by adding an extra penalty term, the algorithm is slowed down, which weakens the superiority of ICD-WT.

**Figure 3:**
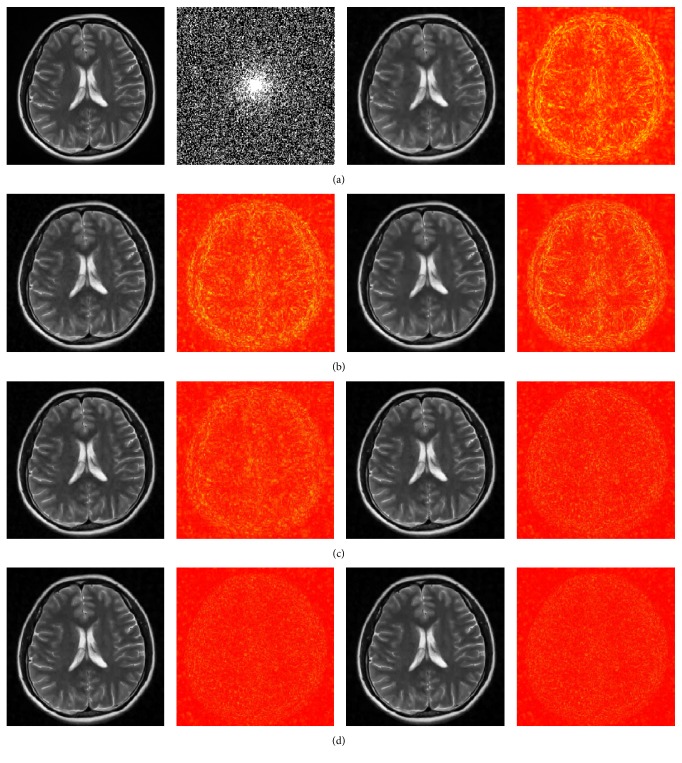
(a) (left to right) shows the original image (Brain-2), the sampling mask, and the reconstructed and error images by SparseMRI. (b) shows the reconstructions by NLTV (left) and ISD-TV (right). (c) shows the reconstructions by SDBS-TV (left) and ICD-TR (right). (d) shows the reconstructions by ICD-TH (left) and ICD-WT (right).

We also tested the proposed ICD on a T2-weighted image (Brain-2) which was acquired from the Siemens scanner 3.0T, SE sequence with imaging parameters: TR = 4000 ms, TE = 91 ms, slice thickness = 5.0 mm, flip angle = 120°, and the field of view (FOV) = 176 × 220 mm × mm. Reconstruction parameters were set as *λ* = 5 × 10^−4^, *L* = 54000, and *w* = 5.  Figure 3 presents the reconstructed images and the error maps of different methods under 30% sampling ratio, while Table 3 gives the HFEN and the SSIM values. RLNE results under different sampling percentages are also given in Table 1. It is straightforward to see that the proposed method performs the best under all sampling ratios.

### 4.3. Parameter Evaluation

**Figure 4:**
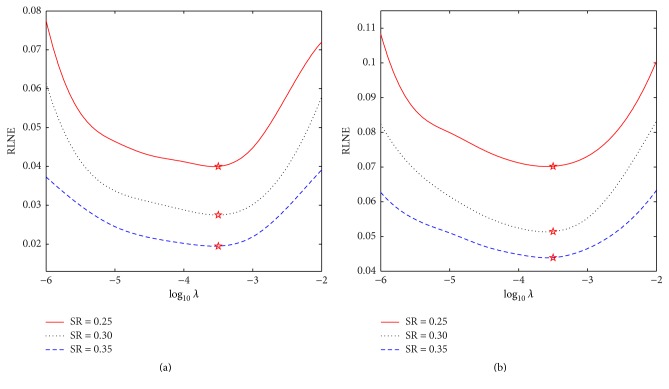
RLNEs versus regularization parameter *λ* for the reconstructions of Brain-1 (a) and Brain-2 (b) under different sampling ratios. Stars indicate the selected *λ*-values.

In this subsection, we explore effects of the parameters involved in the proposed method. We begin by considering the regularization parameter *λ*, since the selection of the optimal *λ*-value is necessary. The reconstructions of Brain-1 and Brain-2 by ICD-TH are performed for different *λ*-values and under different sampling ratios. Motivated by the similar decay rates of four directional transform coefficients, we employed the same *λ* in each direction.  Figure 4 displays the curves of RLNE values as a function of *λ*. The selected *λ*-values are marked with Stars. From the curves, the optimal *λ*-values under different sampling ratios are almost identically selected between 10^−4^~10^−3^.

**Figure 5:**
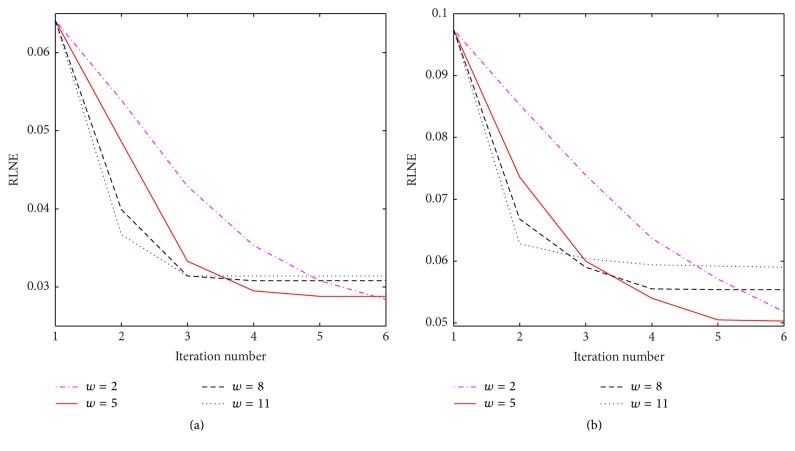
The plots of RLNEs versus iteration number for different choices of *w* on Brain-1 (a) and Brain-2 (b).

Then, we consider effects of the cosupport detection parameters on the performance of the proposed method. As for ICD-TR, we are supposed to evaluate the truncated length *L*.  Table 4 presents the RLNE results of both Brain-1 and Brain-2 under 30% sampling ratio with varying *L*. It can be observed that the proper range of *L* is image-dependent. However, the error undulates slightly when *L* ranges from 52000 to 60000, corresponding to 80~90% of the image dimension. The iterative process makes ICD-TR less sensitive to a few errors present in the cosupport. However with a fixed *L*, the true cosupport is not easy to be identified. We then consider ICD-TH which is more effective and adaptive. The selection of the threshold parameter *w* is essential in that it affects the convergence behavior of the reconstruction. The RLNE between the true and the reconstructed images at each iteration was computed and plotted for different values of *w* = 2,5, 8,11. From the error-iteration curves shown in Figure 5, we see that a large *w* leads to fast convergence but results in a relatively poor reconstruction. This is due to the fact that a large *w* makes the threshold decrease fast so that a number of indices belonging to the true cosupport are excluded from the detected cosupport. On the other hand, a small *w* allows a slowly changing threshold so that the cosupport can be corrected gradually. However, the convergence rate will not be satisfactory provided that *w* is too small. Based on our simulation results, we set *w* = 2~5 to achieve a trade-off between the convergence rate and the reconstruction quality.

The above-mentioned parameter evaluation is based on simulations on a large number of test images. The optimal ranges of the parameters we provided yield good empirical results. However, we realize that the parameters were chosen manually to minimize the reconstruction error. In practical applications, the ground-truth is not available. In these cases, one can conduct parameter selection using the effective L-curve strategy [44] or more sophisticated approaches [45, 46]. The discussion of these approaches is beyond the scope of this paper.

We also tested our method (ICD-TH) on Brain-1 and Brain-2 using radial and Cartesian patterns of 20% and 30% sampling ratios. From the results in Table 5, we see that different patterns may affect the reconstruction quality. However, once the pattern is fixed, our method yields the best performance.

### 4.4. Comparison with Two-Direction Case

In this subsection, we conducted simulations on the Shepp Logan phantom with 12 radial lines, Brain-1 and Brain-2 under 30% sampling ratio, using two-direction ICD.  Table 6 presents the RLNE comparison results, which reveals that the two-direction ICD also performs well. However, compared with four-direction ICD, we see that the local features of the images can be better preserved by adding diagonal components.

### 4.5. Computational Complexity

The proposed method and the methods used for comparison except NLTV were implemented using a nonlinear conjugate gradient method with backtracking line-search. In a MatLab implementation, it took 10 s in average for the proposed ICD-TR and ICD-TH containing only one regularization term, while it took more than 50 s for other methods with an additional wavelet penalty term. However, we expect substantial reduction in the reconstruction time with code optimization and graphics processing unit implementation.

## 5. Conclusion

In this work, we have presented a cosparse analysis model based approach to reconstruct images from highly undersampled data using cosupport constraints. We have demonstrated that the analysis model prior can significantly improve the sparse sampling based image reconstruction. An effective iterative algorithm proceeded by alternating between image reconstruction and cosupport detection. The performance of the proposed method was evaluated through simulations on synthetic and practical MR images. The results indicate that the proposed method yields considerably better performance than methods without using prior knowledge and synthesis model based methods imposing support constraints in terms of both reconstruction accuracy and subjective visual quality.

## Figures and Tables

**Table 1:** RLNE results of test reconstruction methods with random undersampling scheme.

Test image	Sampling ratio	Method						
		SparseMRI	NLTV	ISD-TV	SDBS-TV	ICD-TR	ICD-TH	ICD-WT
Shepp Logan	RL = 10	0.2618	0.1451	0.2338	0.1267	0.0517	0.0390	0.0258
	RL = 11	0.2014	0.1196	0.1852	0.0892	0.0221	0.0117	0.0109
	RL = 12	0.1494	0.0873	0.1009	0.0522	0.0098	0.0042	0.0037

Brain-1	SR = 0.1	0.1773	0.1529	0.1540	0.1480	0.1362	0.1213	0.1131
	SR = 0.2	0.1075	0.0801	0.0803	0.0681	0.0653	0.0601	0.0549
	SR = 0.3	0.0615	0.0385	0.0426	0.0371	0.0309	0.0267	0.0257
	SR = 0.4	0.0346	0.0183	0.0269	0.0178	0.0151	0.0142	0.0139
	SR = 0.5	0.0224	0.0054	0.0184	0.0085	0.0052	0.0047	0.0045

Brain-2	SR = 0.1	0.2036	0.2057	0.1746	0.1681	0.1554	0.1477	0.1436
	SR = 0.2	0.1396	0.1154	0.1016	0.0990	0.0932	0.0875	0.0834
	SR = 0.3	0.0870	0.0710	0.0677	0.0583	0.0550	0.0514	0.0491
	SR = 0.4	0.0587	0.0368	0.0447	0.0411	0.0352	0.0313	0.0302
	SR = 0.5	0.0400	0.0302	0.0321	0.0249	0.0223	0.0206	0.0198

**Table 2:** The number of true/false detections of four directional cosupports over iterations.

Direction	Iteration	Cosupport size	True detections	False detections
Horizontal **Ω**_1_ **x**	1	65074	64036	1038
	2	64601	64022	579
	3	64272	64053	219
	4	64055	64050	5
	5	64054	64054	0

Vertical **Ω**_2_ **x**	1	65103	64472	631
	2	64849	64471	378
	3	64679	64472	207
	4	64473	64472	1
	5	64472	64472	0

Diagonal **Ω**_3_ **x**	1	64993	63705	1288
	2	64374	63691	683
	3	64021	63719	302
	4	63721	63716	5
	5	63720	63720	0

Diagonal **Ω**_4_ **x**	1	64810	63689	1121
	2	64371	63678	693
	3	63998	63707	291
	4	63706	63703	4
	5	63707	63707	0

**Table 3:** HFEN and SSIM results of practical MR images reconstructed using different methods.

Test image	Metric	Method						
		Sparse MRI	NLTV	ISD-TV	SDBS-TV	ICD-TR	ICD-TH	ICD-WT
Brain-1	*ζ*_HFEN_	0.1628	0.0902	0.0971	0.0856	0.0628	0.0514	0.0480
	*ζ*_SSIM_	0.9406	0.9740	0.9703	0.9731	0.9783	0.9851	0.9855

Brain-2	*ζ*_HFEN_	0.1860	0.1547	0.1418	0.1198	0.1034	0.0946	0.0895
	*ζ*_SSIM_	0.8908	0.9061	0.9138	0.9288	0.9357	0.9411	0.9443

**Table 4:** RLNEs with varying truncated length *L* in ICD-TR reconstruction.

Test image	Truncated length *L*(×10^3^)							
	50	52	54	56	58	60	62	64
Brain-1	0.0413	0.0384	0.0355	0.0320	0.0309	0.0336	0.0374	0.0443

Brain-2	0.0633	0.0575	0.0550	0.0563	0.0583	0.0592	0.0612	0.0651

**Table 5:** RLNEs of the reconstructed images using radial and Cartesian *k*-space sampling.

Test image	Method	Radial	Cartesian
Brain-1	SparseMRI	0.1256	0.0833
	NLTV	0.0882	0.0568
	ISD-TV	0.0963	0.0510
	SDBS-TV	0.0771	0.0424
	ICD	0.0669	0.0335

Brain-2	SparseMRI	0.1434	0.0998
	NLTV	0.1249	0.0843
	ISD-TV	0.1138	0.0806
	SDBS-TV	0.1082	0.0712
	ICD	0.0937	0.0608

**Table 6:** Comparison results of RLNEs using two- and four-direction ICD.

Test image	Directional components	Method		
		ICD-TR	ICD-TH	ICD-WT
Shepp Logan	Two	0.0254	0.0205	0.0169
	Four	0.0098	0.0042	0.0037

Brain-1	Two	0.0388	0.0354	0.0321
	Four	0.0309	0.0267	0.0257

Brain-2	Two	0.0661	0.0627	0.0607
	Four	0.0550	0.0514	0.0491

## Appendix

### Proof of Proposition 3

In this part, we first prove the following two lemmas that are the key elements for the proof of Proposition 3. Note that our goal is to quantify the upper bound:

(A.1) maxΛ dimWΛmaxΛV−VΛ+JΛ=V−minΛVΛ−JΛ.

To simplify the problem, we look at an equivalent quantity:

(A.2) $$\begin{array}{c} \alpha \left(\;\Lambda \;\right)≔\underset{\Lambda }{min}\left\{\;\left|\;V\left(\;\Lambda \;\right)\;\right|-J\left(\;\Lambda \;\right)\;\right\}. \end{array}$$

Lemma A.1 A.1. Given the known part of the cosupport Λ_0_ with *J* connected components, then for a fixed cosparsity level *l*, the value *α*(Λ) = min_Λ_{|*V*(Λ)| − *J*(Λ)} is achieved when *J*(Λ) = *J*.

Proof If *J*(Λ) > *J*, there exist extra connected components out of *V*(Λ_0_). Without loss of generality, we only consider the case where *J*(Λ∖Λ_0_) = 1. Let Λ^1^ ⊂ Λ∖Λ_0_ correspond to the extra component. Thus, we have *J*(Λ) = *J* + 1. Notice that a subgraph can be shifted horizontally or vertically unless it has vertices on all four boundaries of *V*. Since Λ^1^ and Λ_0_ are disconnected, we can shift Λ^1^ towards Λ_0_ until they first met. Suppose *t* and *s* are the numbers of the vertices and the edges coincided. Clearly, *s* ≤ *t* − 1. The resulting subgraph Λ^2^ has |*V*(Λ)| − *t* vertices, |Λ | − *s* edges, and *J* connected components. Then, we add *s* additional edges that are connected to Λ^2^, thereby yielding Λ^3^. From this we can infer that |Λ^3^ | = |Λ | = *l*, *J*(Λ^3^) = *J*, and

(A.3) $$\begin{array}{c} \left|\;V\left(\;\Lambda^{3}\;\right)\;\right|\leq \left|\;V\left(\;\Lambda^{2}\;\right)\;\right|+s=\left|\;V\left(\;\Lambda \;\right)\;\right|-t+s. \end{array}$$

We further have that

(A.4) VΛ3−JΛ3VΛ−t+s−JΛ+1≤VΛ−JΛ.

Consequently, we obtain Λ^3^ from Λ that satisfies |Λ^3^ | = *l* and *J*(Λ^3^) = *J*. However, a smaller *α*(Λ) is achieved, which completes the proof. One can consider cases in which *J*(Λ∖Λ_0_) > 1 in the same vein.

Lemma A.1 implies that quantifying the upper bound in (A.1) is equivalent to quantifying the value min_|Λ|=*l*_ | *V*(Λ)|. In the next lemma, we provide a lower bound for |*V*(Λ)| and prove that the value is achieved when all the *J* connected components share the same cosparsity.

Lemma A.2 A.2. For a fixed *l*, the value

(A.5) $$\begin{array}{c} \left|\;V\left(\;\Lambda \;\right)\;\right|\geq \frac{l}{2}+\frac{J}{2}+{\left(\;\frac{Jl}{2}+\frac{J^{2}}{4}\;\right)}^{1/2}. \end{array}$$

Proof We first denote the edge sets of the *J* connected components by Λ_*i*_ ⊂ Λ with |Λ_*i*_ | = *l*_*i*_ (*i* ∈ *I* = {1,…, *J*}). Clearly, we have ∑_*i*_ *l*_*i*_ = *l*. Then, we enlarge each edge set Λ_*i*_ as

(A.6) $$\begin{array}{c} {\bar{\Lambda }}_{i}=\left\{\;e_{↓}\left(\;v\;\right),e_{\to }\left(\;v\;\right):v\in V\left(\;\Lambda_{i}\;\right)\;\right\}, \end{array}$$

where *e*_↓_(*v*) (resp., *e*_→_(*v*)) denotes the edge extending downwards (resp., rightwards) from the vertex *v*. For any *i* ∈ *I*, we have $\left|{\bar{\Lambda }}_{i}|=2|V(\Lambda_{i})\right|$. According to [17, Lemma 15], the value min_|Λ_*i*_|=*l*_*i*__ | *V*(Λ_*i*_)| occurs when *V*(Λ_*i*_) is square, and the optimal width of *V*(Λ_*i*_) is given as

(A.7) $$\begin{array}{c} w_{i}=\frac{1}{2}+{\left(\;\frac{l_{i}}{2}+\frac{1}{4}\;\right)}^{1/2}. \end{array}$$

Then, we have

(A.8) $$\begin{array}{c} 2\left|\;V\left(\;\Lambda_{i}\;\right)\;\right|=\left|\;{\bar{\Lambda }}_{i}\;\right|\geq l_{i}+2w_{i}. \end{array}$$

Summing the above over all *i* ∈ *I* yields

(A.9) 2VΛ∑i∈I2VΛi≥∑i∈Ili+2∑i∈I12+li2+141/2=l+J+∑i∈I2li+11/2.

For any *j* ∈ *I*,

(A.10) $$\begin{array}{c} l_{j}=l-\underset{i\in I\setminus j}{\sum }l_{i}. \end{array}$$

Substituting (A.10) into (A.9),

(A.11) 2VΛl+J+∑i∈I∖j2li+11/2+2lj+11/2=l+J+∑i∈I∖j2li+11/2+2l−∑i∈I∖jli+11/2.

Then for any *i* ≠ *j*, the minimum of the right hand side of (A.11) is achieved at *l*_*i*_ = *l* − ∑_*i*∈*I*∖*j*_ *l*_*i*_ = *l*_*j*_. This reveals that all the *J* connected components share the same cosparsity. Therefore, by denoting *l*_*i*_ = *l*/*J*  (*i* ∈ *I*) and substituting into (A.9), we conclude that

(A.12) $$\begin{array}{c} \left|\;V\left(\;\Lambda \;\right)\;\right|\geq \frac{l}{2}+\frac{J}{2}+{\left(\;\frac{Jl}{2}+\frac{J^{2}}{4}\;\right)}^{1/2} \end{array}$$

as required.

Proof of Proposition 3. The proof of Proposition 3 is trivial by applying Lemmas A.1 and A.2 to (A.1):

(A.13) maxΛ dimWΛV−minΛVΛ−JΛ=n−minΛVΛ+J=n−l2−J2−Jl2+J241/2+J≤n−l2−Jl2+J2,

which leads to the minimum measurement number:

(A.14) $$\begin{array}{c} m\geq 2n-l-\sqrt{2Jl}+J\geq 2\underset{\Lambda }{max}\;dim\left(\;W_{\Lambda }\;\right). \end{array}$$

The proof is then completed.
